# Risk of tobacco-related multiple primary cancers in Bavaria, Germany

**DOI:** 10.1186/1471-2407-12-250

**Published:** 2012-06-18

**Authors:** Ulrike Braisch, Martin Meyer, Martin Radespiel-Tröger

**Affiliations:** 1 Population-Based Cancer Registry Bavaria, Östliche Stadtmauerstr. 30, 91054, Erlangen, Germany

## Abstract

**Background:**

With the prospect of increasing prevalence of cancer, the issue of multiple primary cancers becomes more relevant. The aim of this study was to estimate the risk of developing a tobacco-related subsequent primary cancer (TRSPC) in persons with a tobacco-related first primary cancer (TRFPC) compared with the general population in Bavaria, Germany.

**Methods:**

Using data from the Population-Based Cancer Registry Bavaria, we analyzed TRFPC and TRSPC diagnosed in Bavaria between 2002 and 2008 to estimate the relative and absolute risk of developing TRSPC using standardized incidence ratios (SIR) and excess absolute risks (EAR).

**Results:**

121,631 TRFPC in men and 75,886 respective cancers in women were registered, which in 2.5% of male and 1.2% of female cancer patients were followed by at least one TRSPC. In both males and females, the highest increased risks compared to the general population were found within the group of cancer in the mouth/pharynx, oesophagus, larynx, and lung/bronchus.

**Conclusions:**

With respect to cancer in the mouth/pharynx, oesophagus, larynx, lung/bronchus, kidney, urinary bladder and urinary tract, smoking was confirmed as a shared risk factor based on our finding of mutually significantly increased risks of TRSPC. The results of this study illustrate the importance of smoking cessation and of continued follow-up care especially of smokers with the aforementioned TRFPC to detect TRSPC at an early stage.

## Background

Probably due in part to technological advances in the early detection and treatment of cancer, the prevalence of cancer in Germany is rising [1]. Therefore, interest has increased with regard to the risk of developing subsequent primary cancers in cancer survivors.

Tobacco smoking is one of the most easily preventable risk factors for cancer. It is responsible for about 20% of all cancer deaths in Germany [2]. In view of the public health-relevance of tobacco-related cancer, it is important to estimate the burden of tobacco-related subsequent primary cancer (TRSPC) among persons with tobacco-related first primary cancer (TRFPC).

Therefore, in this population-based study, we analyzed tobacco-related cancers. According to Schottenfeld [3], this group includes cancer of the mouth and pharynx, oesophagus, stomach, colon and rectum, pancreas, larynx, lungs and bronchus, kidneys, urinary tract, urinary bladder, respectively, and leukemia. For malignancies in the mouth/pharynx, oesophagus, pancreas, larynx, lung/bronchus, kidney, urinary tract, and urinary bladder, tobacco smoking is considered as a main risk factor. For malignancies in the stomach, colon/rectum as well as for leukemia, a weaker but positive association with tobacco smoking is known or at least suspected [3].

With regard to the aforementioned malignancies, the aim of this study was to estimate the risk of developing TRSPC among persons with TRFPC compared with the general population, and to test the etiologic hypothesis that the risk of TRSPC is significantly increased among persons with TRFPC because of the shared risk factor tobacco smoking. Furthermore, based on our results, it may be possible to obtain new insights into the etiology of the evaluated cancer types.

## Methods

In the federal state of Bavaria, located in the southeast of Germany, population-based cancer registration commenced in 1998, and full-scale registration covering a population of approx. 12.4 million started in 2002. All doctors, dentists, pathologists, and local health authorities are asked to send notifications about all types of newly diagnosed cancer to their respective regional cancer centre where they are collected. Notifications are forwarded to the confidentiality office of the Bavarian cancer registry in Nuremberg for the purpose of data checking and pseudonymization. All pseudonymized notifications are sent to the Bavarian cancer registration office in Erlangen where they are linked by means of stochastic record linkage [4], stored permanently, and analysed.

In this study we analyzed TRFPC and TRSPC diagnosed in Bavaria between 2002 and 2008 in persons aged 15 years or older, utilizing data from the Population Based Cancer Registry Bavaria. Cases of childhood cancer were excluded from the evaluation because of the different classification of childhood cancer [5]. DCO (death certificate only) cases were included in these analyses.

The date of diagnosis was defined by the month and year of the diagnosis because the day of diagnosis is not recorded in this registry. Using the methodology proposed by Schoenberg and Myers [6], 2,934 patients (1.5%) were excluded from the evaluation because the diagnoses of TRFPC and TRSPC, respectively, were made at the same date. After this exclusion, 121,631 TRFPC in men and 75,886 respective cancers in women were included in the analysis.

Cancer diagnoses were coded according to the International Classification of Diseases 10^th^ Revision (ICD-10) [7].

We report on the risk of TRSPC among persons with TRFPC compared with the general population. This group includes cancer in the mouth/pharynx (ICD-10: C00-C14), oesophagus (C15), stomach (C16), colon/rectum (C18-C21), pancreas (C25), larynx (C32), bronchus/lung (C33-C34), kidney (C64), urinary tract (C65-C66, C68), and the urinary bladder (C67, D09.0, D41.4), respectively, and leukemia (C91-C95) [3].

Except for the diagnosis group of the urinary bladder (C67, D09.0, D41.4), only invasive carcinomas were examined in accordance with published guidelines of cancer registration [8]. In the urinary bladder, only the first diagnosed urinary bladder tumour was counted regardless of its invasiveness [8].

A commonly used estimator for the relative risk of a subsequent primary cancer among cancer patients compared with the general population is the standardized incidence ratio (SIR) calculated according to Schoenberg and Myers [6], which compares the number of observed cases with the expected number of cases calculated according to the method of indirect standardization by taking into account the different age distributions in the study population and in the reference population, respectively [9], and the year of diagnosis.

In order to estimate the risk of TRSPC, we need to calculate the individual times at risk (person-years at risk, PY) of developing a subsequent malignancy among cancer patients with a TRFPC. The individual PY were calculated as the time from the diagnosis of the first cancer until the date of death or December 2008, whichever occurred first.

Data were stratified by sex, site of first primary cancer, site of subsequent primary cancer, 5-year age groups, and calendar year. The expected number of subsequent primary cancers in each stratum was obtained by multiplying the sex-, site-, age-, and calendar year-specific incidence rates (rates per person-year) by the related person-years at risk. These cases were then added up for all age groups and calendar years to obtain the total number of expected cases at each site for each sex.

The exact 95% confidence interval (95% CI) of the SIR was calculated according to Garwood [10] assuming a Poisson distribution of the observed number of cases. This method is also suggested by Sahai *et al.*[11] and SEER [12]. An increased or decreased risk is considered to be statistically significant if the associated confidence interval does not include the value one.

The SIR is commonly used to test etiologic hypotheses, e.g. the hypothesis of a significantly increased risk of TRSPC among persons with TRFPC. In this context, the hypothesis of a risk factor shared between two malignancies may be confirmed if the risk is mutually and significantly increased, that is, if both tumour A after tumour B and tumour B after tumour A show a significantly increased risk of subsequent malignancy among cancer patients compared to the general population [13].

A second useful measure is the excess absolute risk (EAR), which is computed by subtracting the number of expected cases from the number observed cases, dividing this difference by the sum of observed person-years and then multiplying by 10,000 to obtain the excess of cases in cancer patients compared with the general reference population per 10,000 person-years [13,14]. The EAR is often used to measure the burden of a specific subsequent primary cancer in the population of defined cancer patients. It is reported in this paper in addition to the SIR for each first and subsequent site of cancer according to sex.

The SIR and EAR provide different information about the risk of developing subsequent malignancies among cancer patients compared with the general population and therefore complement each other. For example, in rare tumours, it may happen that there are three observed cases of a specific subsequent cancer in a defined cancer population but only one expected case. In this case, the SIR would show a threefold risk increase while the absolute excess would be only 2 cases, which would result in a low EAR. This example demonstrates the importance of EAR for determining the need of follow-up examinations among cancer patients because it provides information on the relative usefulness of these examinations compared to other possible interventions.

Because all personal data were pseudonymised prior to analysis and only aggregated results are published, no ethical review committee approval was required for the purpose of the present observational study which was performed in compliance with the Declaration of Helsinki [15].

All analyses were conducted using a self-programmed software written in the statistical programming language R [16].

## Results

During the follow-up period (median, 1.0/0.8 years in men/women), 3,097 (2.5%) male and 924 (1.2%) female cancer patients experienced at least one TRSPC.

In Table 1, the numbers of included patients, their median age at diagnosis of first primary cancer, their total and their median person-years at risk are given according to sex and site of TRFPC, respectively. Except for pancreatic cancer, the number of patients was greater in men than in women. The median age at diagnosis of first primary cancer was higher in women than in men, except for cancer in the larynx and lung/bronchus. The median follow-up times were similar among men and women.

**Table 1:** Patient characteristics (number of persons, total person-years at risk of subsequent primary cancer, median age at diagnosis of first primary cancer, and median person-years at risk of subsequent primary cancer according to site of first tobacco-related primary cancer and sex; Bavaria; 2002 – 2008)

	**Men**				**Women**			
**Site of first primary cancer**	**n**	**Median age**	**Sum PY**	**Median PY**	**n**	**Median age**	**Sum PY**	**Median PY**
mouth and pharynx	8,579	60	17,901	1.4	2,673	64	5,942	1.7
oesophagus	4,084	65	4,759	0.6	959	70	983	0.5
stomach	9,613	72	12,593	0.6	7,896	78	9,810	0.5
colon and rectum	34,663	70	74,122	1.7	29,262	75	58,642	1.4
pancreas	6,961	70	4,183	0.3	7,364	76	3,992	0.2
larynx	2,916	64	6,989	1.9	356	63	829	1.8
bronchus and lung	25,733	69	22,923	0.3	11,087	68	11,301	0.5
kidney	7,760	67	17,985	1.8	5,192	72	11,581	1.8
urinary tract	1,135	73	1,923	1.0	795	76	1,429	1.2
urinary bladder	14,726	72	35,950	2.0	5,654	76	12,241	1.5
leukemia	5,461	69	9,116	0.8	4,648	74	6,565	0.3
**all cancer sites combined**	**121,631**	**69**	**208,444**	**1.0**	**75,886**	**74**	**123,315**	**0.8**

n, number of persons; median age, median age at diagnosis of first primary cancer; PY, person-years at risk of subsequent primary cancer; sum PY, total person-years at risk of subsequent primary cancer; median PY, median person-years at risk of subsequent primary cancer.

The relative and absolute risks of TRSPC are given in Table 2 according to sex and type of TRFPC and TRSPC, respectively.

**Table 2:** Relative and absolute risks of subsequent tobacco-related primary cancer according to type of first tobacco-related primary cancer and sex (Bavaria; 2002–2008)

			**Men**		**Women**		
**First tobacco-related primary cancer**	**Subsequent tobacco-related primary cancer**	**O**	**SIR (95% CI)**	**EAR**	**O**	**SIR (95% CI)**	**EAR**
mouth, pharynx	oesophagus	86	**19.63** (15.71-24.25)	45.6	15	**48.06** (26.9-79.27)	24.7
	stomach	24	**2.87** (1.84-4.27)	8.7	2	0.93 (0.11-3.35)	−0.3
	colon, rectum	54	**1.65** (1.24-2.15)	11.9	12	1.42 (0.74-2.49)	6.0
	pancreas	9	1.36 (0.62-2.58)	1.3	2	0.92 (0.11-3.31)	−0.3
	larynx	43	**14.10** (10.21-18.99)	22.3	7	**69.05** (27.76-142.26)	11.6
	bronchus, lung	201	**7.90** (6.85-9.07)	98.1	42	**12.36** (8.91-16.71)	65.0
	kidney	17	**2.11** (1.23-3.38)	5.0	2	1.27 (0.15-4.60)	0.7
	urinary tract	3	2.82 (0.58-8.24)	1.1	-	-	-
	urinary bladder	18	1.28 (0.76-2.02)	2.2	2	1.18 (0.14-4.28)	0.5
	leukemia	14	**3.03** (1.66-5.09)	5.2	2	1.59 (0.19-5.75)	1.25
oesophagus	mouth, pharynx	36	**14.18** (9.93-19.64)	70.3	5	**34.92** (11.34-81.48)	49.4
	stomach	5	1.64 (0.53-3.82)	4.1	1	2.41 (0.06-13.42)	6.0
	colon, rectum	23	**1.99** (1.26-2.98)	24.0	6	**3.70** (1.36-8.05)	44.5
	pancreas	5	2.15 (0.70-5.01)	5.6	2	4.71 (0.57-17.00)	16.0
	larynx	7	**7.44** (2.99-15.33)	12.7	-	-	-
	bronchus, lung	23	**2.62** (1.66-3.92)	29.9	6	**9.42** (3.46-20.50)	54.5
	kidney	8	**2.97** (1.28-5.86)	11.2	2	6.54 (0.79-23.62)	17.2
	urinary tract	1	2.53 (0.06-14.12)	1.3	-	-	-
	urinary bladder	12	**2.34** (1.21-4.09)	14.4	1	3.07 (0.08-17.13)	6.9
	leukemia	6	**3.65** (1.34-7.94)	9.2	-	-	-
stomach	mouth, pharynx	6	0.97 (0.36-2.12)	−0.1	1	0.70 (0.02-3.91)	−0.4
	oesophagus	8	2.07 (0.90-4.09)	3.3	1	1.56 (0.04-8.71)	0.4
	colon, rectum	68	**1.68** (1.30-2.13)	21.8	40	**1.86** (1.33-2.54)	18.9
	pancreas	17	**2.09** (1.22-3.34)	7.0	13	**2.24** (1.19-3.83)	7.3
	larynx	3	1.18 (0.24-3.43)	0.4	-	-	-
	bronchus, lung	33	1.15 (0.79-1.62)	3.5	9	1.34 (0.61-2.54)	2.3
	kidney	19	**2.26** (1.36-3.53)	8.4	6	1.66 (0.61-3.62)	2.4
	urinary tract	1	0.65 (0.02-3.64)	−0.4	-	-	-
	urinary bladder	17	0.87 (0.51-1.39)	−2.0	3	0.69 (0.14-2.01)	−1.4
	leukemia	11	1.80 (0.90-3.22)	3.9	6	1.88 (0.69-4.09)	2.9
colon, rectum	mouth, pharynx	56	**1.48** (1.12-1.92)	2.5	13	1.50 (0.80-2.56)	0.7
	oesophagus	31	1.33 (0.91-1.89)	1.1	8	2.07 (0.89-4.08)	0.7
	stomach	73	1.11 (0.87-1.40)	1.0	45	1.31 (0.96-1.75)	1.8
	pancreas	43	0.92 (0.66-1.23)	−0.5	44	1.30 (0.94-1.74)	1.7
	larynx	15	0.97 (0.54-1.60)	−0.1	-	-	-
	bronchus, lung	210	**1.26** (1.09-1.44)	5.8	60	**1.47** (1.12-1.89)	3.3
	kidney	85	**1.71** (1.37-2.12)	4.8	36	**1.67** (1.17-2.31)	2.5
	urinary tract	12	1.40 (0.72-2.44)	0.5	7	1.86 (0.75-3.84)	0.6
	urinary bladder	148	**1.34** (1.13-1.58)	5.1	38	**1.49** (1.06-2.05)	2.1
	leukemia	59	**1.71** (1.3-2.21)	3.3	32	**1.73** (1.18-2.44)	2.3
pancreas	mouth, pharynx	1	0.47 (0.01-2.59)	−2.7	1	1.77 (0.04-9.87)	1.1
	oesophagus	1	0.80 (0.02-4.48)	−0.6	-	-	-
	stomach	9	**2.91** (1.33-5.52)	14.1	7	**3.60** (1.45-7.42)	12.7
	colon, rectum	16	1.41 (0.81-2.29)	11.1	12	1.63 (0.84-2.84)	11.6
	larynx	3	3.57 (0.74-10.43)	5.2	-	-	-
	bronchus, lung	10	1.18 (0.57-2.18)	3.7	3	1.12 (0.23-3.26)	0.8
	kidney	4	1.58 (0.43-4.04)	3.5	4	2.88 (0.79-7.39)	6.6
	urinary bladder	7	1.36 (0.55-2.81)	4.5	-	-	-
	leukemia	1	0.61 (0.02-3.40)	−1.5	2	1.82 (0.22-6.58)	2.26
larynx	mouth, pharynx	33	**8.83** (6.08-12.41)	41.9	3	**26.52** (5.47-77.51)	34.8
	oesophagus	18	**8.92** (5.28-14.09)	22.9	1	23.53 (0.60-131.09)	11.6
	stomach	5	1.10 (0.36-2.57)	0.7	1	3.95 (0.10-22.03)	9.0
	colon, rectum	18	1.05 (0.63-1.67)	1.3	-	-	-
	pancreas	9	**2.61** (1.19-4.95)	7.9	1	3.74 (0.09-20.82)	8.8
	bronchus, lung	69	**5.37** (4.18-6.80)	80.4	9	**18.83** (8.61-35.75)	102.9
	kidney	6	1.53 (0.56-3.32)	3.0	-	-	-
	urinary tract	1	1.71 (0.04-9.53)	0.6	-	-	-
	urinary bladder	10	1.31 (0.63-2.41)	3.4	1	4.79 (0.12-26.68)	9.6
	leukemia	2	0.82 (0.10-2.97)	−0.6	-	-	-
bronchus, lung	mouth, pharynx	42	**3.48** (2.51-4.71)	13.1	10	**6.46** (3.10-11.88)	7.5
	oesophagus	20	**2.83** (1.73-4.37)	5.6	3	4.84 (1.00-14.14)	2.1
	stomach	28	**1.60** (1.06-2.31)	4.6	12	**2.88** (1.49-5.03)	6.9
	colon, rectum	71	1.10 (0.86-1.39)	2.8	17	1.02 (0.59-1.63)	0.3
	pancreas	19	1.47 (0.89-2.30)	2.7	10	**2.31** (1.11-4.24)	5.0
	larynx	37	**7.78** (5.48-10.72)	14.1	1	4.94 (0.12-27.50)	0.7
	kidney	32	**2.21** (1.51-3.12)	7.6	13	**3.94** (2.10-6.74)	8.6
	urinary tract	6	2.62 (0.96-5.71)	1.6	3	**5.93** (1.22-17.33)	2.2
	urinary bladder	63	**2.15** (1.65-2.75)	14.7	9	**2.69** (1.23-5.11)	5.0
	leukemia	16	1.72 (0.98-2.80)	2.9	3	1.21 (0.25-3.53)	0.5
kidney	mouth, pharynx	10	1.11 (0.53-2.03)	0.5	1	0.62 (0.02-3.44)	−0.5
	oesophagus	4	0.76 (0.21-1.95)	−0.7	-	-	-
	stomach	15	1.14 (0.64-1.89)	1.1	5	0.90 (0.29-2.09)	−0.5
	colon, rectum	51	1.06 (0.79-1.39)	1.5	25	1.17 (0.76-1.73)	3.2
	pancreas	9	0.93 (0.42-1.76)	−0.4	5	0.87 (0.28-2.02)	−0.7
	larynx	3	0.86 (0.18-2.50)	−0.3	1	5.08 (0.13-28.28)	0.7
	bronchus, lung	54	**1.51** (1.13-1.97)	10.1	16	**2.06** (1.18-3.34)	7.1
	urinary tract	3	1.75 (0.36-5.12)	0.7	3	4.47 (0.92-13.07)	2.0
	urinary bladder	51	**2.31** (1.72-3.03)	16.1	10	**2.29** (1.10-4.21)	4.9
	leukemia	12	1.72 (0.89-3.00)	2.8	2	0.63 (0.08-2.28)	−1.0
urinary tract	oesophagus	1	1.64 (0.04-9.15)	2.0	-	-	-
	stomach	2	1.05 (0.13-3.80)	0.5	-	-	-
	colon, rectum	12	1.84 (0.95-3.21)	28.5	4	1.21 (0.33-3.10)	4.9
	pancreas	3	2.28 (0.47-6.66)	8.8	3	3.35 (0.69-9.78)	14.7
	bronchus, lung	9	1.96 (0.89-3.72)	22.9	4	**3.82** (1.04-9.77)	20.7
	kidney	4	2.97 (0.81-7.60)	13.8	-	-	-
	urinary bladder	5	1.58 (0.51-3.68)	9.5	12	**17.84** (9.22-31.15)	79.3
	leukemia	2	2.03 (0.25-7.32)	5.3	-	-	-
urinary bladder	mouth, pharynx	25	1.41 (0.91-2.08)	2.0	3	1.63 (0.34-4.77)	1.0
	oesophagus	12	1.05 (0.54-1.83)	0.2	3	3.60 (0.74-10.53)	1.8
	stomach	41	1.13 (0.81-1.53)	1.3	16	**2.10** (1.20-3.40)	6.8
	colon, rectum	144	1.16 (0.98-1.36)	5.5	26	0.95 (0.62-1.39)	−1.2
	pancreas	31	1.24 (0.84-1.75)	1.7	6	0.80 (0.29-1.74)	−1.2
	larynx	4	0.53 (0.15-1.36)	−1.0	-	-	-
	bronchus, lung	187	**2.16** (1.86-2.49)	27.9	25	**2.87** (1.85-4.23)	13.3
	kidney	77	**3.04** (2.40-3.80)	14.4	12	**2.56** (1.32-4.48)	6.0
	urinary tract	7	1.47 (0.59-3.03)	0.6	6	**7.26** (2.66-15.79)	4.2
	leukemia	30	**1.59** (1.07-2.27)	3.1	8	1.96 (0.85-3.87)	3.2
leukemia	mouth, pharynx	3	0.73 (0.15-2.13)	−1.2	2	2.42 (0.29-8.73)	1.8
	oesophagus	5	2.10 (0.68-4.89)	2.9	-	-	-
	stomach	9	1.43 (0.66-2.72)	3.0	4	1.50 (0.41-3.84)	2.0
	colon, rectum	27	1.19 (0.79-1.74)	4.8	14	1.37 (0.75-2.30)	5.8
	pancreas	6	1.32 (0.48-2.87)	1.6	4	1.48 (0.40-3.78)	2.0
	larynx	1	0.63 (0.02-3.48)	−0.7	-	-	-
	bronchus, lung	27	**1.63** (1.08-2.38)	11.5	7	1.83 (0.74-3.77)	4.8
	kidney	7	1.40 (0.56-2.89)	2.2	-	-	-
	urinary tract	3	3.68 (0.76-10.76)	2.4	-	-	-
	urinary bladder	11	1.05 (0.52-1.87)	0.5	4	1.94 (0.53-4.97)	3.0

O, number of observed cases; SIR, standardized incidence ratio; 95% CI, 95% confidence interval; EAR, excess absolute risk per 10,000; bold type, SIR significantly different from one.

We found considerable differences between men and women in view of the magnitude of the estimated relative and absolute risk of TRSPC.

With regard to first primary cancers in mouth/pharynx, oesophagus, larynx and urinary tract, SIRs were considerably higher in women than in men, whereas in about half of these analyses, the corresponding EAR was lower in women than in men. The largest SIR was observed in women with subsequent primary cancer (SPC) in the larynx after first primary cancer (FPC) in the mouth/pharynx (SIR, 69.05; O = 7), whereas the corresponding SIR among men was only 14.10 (O = 43). The corresponding EAR, however, in men (22 cases per 10,000 person years) was about twice as high as in women. Among men, the largest *relative* risk was found with SPC in the oesophagus after FPC in mouth/pharynx (SIR, 19.63). The largest *absolute* risk, however, was observed among men for SPC in the lung/bronchus after FPC in the mouth/pharynx (EAR, 98.1) and among women for SPC in the lung/bronchus after FPC in the larynx (EAR, 102.9).

Based on the presented results, we found five groups of TRFPC with mutually and significantly increased risk of TRSPC each of which was identified among at least one sex, and which are presented in Table 3 by splitting the groups into dyads. Furthermore, known shared risk factor(s) are reported.

**Table 3:** **Groups of tobacco-related cancer sites with mutually significantly increased risk of subsequent primary cancer valid for at least one sex and split into pairs (Bavaria; 2002–2008; for each pair, the respective identified sex is given as well as known shared risk factors; data sources,**[17-21]**)**

**Group**	**Pair of cancer types with mutually significantly elevated risk of subsequent primary cancer**	**Sex**	**Shared risk factors in addition to tobacco smoking**
1	mouth/pharynx – oesophagus	m,f	alcohol consumption, low fruit and vegetable intake, low socioeconomic status
	mouth/pharynx – larynx	m,f	alcohol consumption, low fruit and vegetable intake
	mouth/pharynx – lung/bronchus	m,f	low fruit and vegetable intake
	oesophagus – larynx	m	alcohol consumption, low fruit and vegetable intake
	larynx – lung/bronchus	m	low fruit and vegetable intake
	lung/bronchus – oesophagus	m	low fruit and vegetable intake
2	lung/bronchus – kidney	m,f	-
	kidney – bladder	m,f	-
	bladder – lung/bronchus	m,f	-
3	bladder – lung/bronchus	m,f	-
	lung/bronchus – urinary tract	f	-
	urinary tract – bladder	f	ionizing radiation, heavy use of phenacetin-containing analgesics
4	mouth/pharynx – colon/rectum	m	alcohol consumption, low fruit and vegetable intake
5	stomach – pancreas	m,f	obesity

Group 1 contained malignancies in the mouth/pharynx (C00-C14), oesophagus (C15), larynx (C32), and lung/bronchus (C33-C34). However, all pairwise associations within this group revealed a mutually significantly increased risk only among men. Among women, mutually significantly SIRs were only found in three dyads: (1) mouth/pharynx and oesophagus; (2) mouth/pharynx and larynx; (3) mouth/pharynx and lung/bronchus.

Group 2 was identified in both sexes and included primary cancers in the lung/bronchus, in the kidneys (C64), and in the urinary bladder (C67 + D09.0 + D41.4).

Similarly, group 3 contained the dyad of urinary bladder and lung/bronchus, but in addition also the dyads lung/bronchus with the urinary tract (C65-C66, C68), and the urinary tract with the urinary bladder. However, this group was only found to exist among women.

Group 4 and 5, respectively, incorporate only two types of cancer each: group 4, malignancies in the mouth/pharynx and colon/rectum (C18-C21), respectively; group 5, malignancies in the stomach (C16) and pancreas (C25). Both groups were identified in men. Among women, however, only group 5 was found to exist.

In Table 4, the frequency distribution of treatment in Bavaria is given according to site of first primary cancer as seen in our data. The most frequent treatment was surgery except in leukemia which is predominantly treated by chemotherapy.

**Table 4:** Frequency distribution of treatment according to site of first primary cancer (Bavaria; 2002–2008; DCO cases excluded; treatments are grouped so that at least the three most frequent treatments of each cancer site are shown)

	**Treatment**													
**Site of first primary cancer**	**Surg**	**Radio**	**Chemo**	**Other therapy**	**Surg + radio**	**Surg + chemo**	**Surg + other therapy**	**Radio + chemo**	**Chemo + other therapy**	**Surg + radio + chemo**	**Other treatment**	**No treatment**	**Missing**	**Row sum**
mouth, pharynx	**2,844**	430	78	380	**1,762**	82	422	1,016	28	**1,192**	484	219	1,391	10,328
	**(28%)**	(4%)	(1%)	(4%)	**(17%)**	(1%)	(4%)	(10%)	(0%)	**(12%)**	(5%)	(2%)	(14%)	(100%)
oesophagus	**764**	203	232	**350**	46	167	154	**495**	66	232	271	204	985	4,169
	**(18%)**	(5%)	(6%)	**(8%)**	(1%)	(4%)	(4%)	**(12%)**	(2%)	(6%)	(7%)	(5%)	(24%)	(100%)
stomach	**6,519**	51	**751**	160	39	**1,192**	249	65	92	274	234	677	3,041	13,344
	**(49%)**	(0%)	**(6%)**	(1%)	(0%)	**(9%)**	(2%)	(1%)	(1%)	(2%)	(2%)	(5%)	(23%)	(100%)
colon, rectum	**29,367**	223	342	567	656	**7,861**	2,013	623	56	**3,927**	1,855	783	5,189	53,462
	**(55%)**	(0%)	(1%)	(1%)	(1%)	**(15%)**	(4%)	(1%)	(0%)	**(7%)**	(4%)	(2%)	(10%)	(100%)
pancreas	**1,837**	40	**1,430**	232	39	**1,134**	167	159	198	185	305	393	1,946	8,065
	**(23%)**	(1%)	**(18%)**	(3%)	(1%)	**(14%)**	(2%)	(2%)	(3%)	(2%)	(4%)	(5%)	(24%)	(100%)
larynx	**1,359**	121	21	41	**357**	21	69	129	6	**228**	76	64	397	2,889
	**(47%)**	(4%)	(1%)	(1%)	**(12%)**	(1%)	(2%)	(5%)	(0%)	**(8%)**	(3%)	(2%)	(14%)	(100%)
bronchus, lung	**4,538**	1,876	**4,506**	579	825	1,294	226	**3,072**	640	957	1,588	1,428	5,617	27,146
	**(17%)**	(7%)	**(17%)**	(2%)	(3%)	(5%)	(1%)	**(11%)**	(2%)	(4%)	(6%)	(5%)	(21%)	(100%)
kidney	**9,013**	46	30	55	**140**	135	**165**	3	16	35	238	93	875	10,844
	**(83%)**	(0%)	(0%)	(1%)	**(1%)**	(1%)	**(2%)**	(0%)	(0%)	(0%)	(2%)	(1%)	(8%)	(100%)
urinary tract	**1,119**	3	7	7	**41**	**148**	29	_	2	27	15	23	149	1,570
	**(71%)**	(0%)	(0%)	(0%)	**(3%)**	**(9%)**	(2%)		(0%)	(2%)	(1%)	(2%)	(10%)	(100%)
urinary bladder	**13,306**	13	17	16	219	**1,562**	**467**	10	4	234	172	206	2,226	18,452
	**(72%)**	(0%)	(0%)	(0%)	(1%)	**(9%)**	**(3%)**	(0%)	(0%)	(1%)	(1%)	(1%)	(12%)	(100%)
leukemia	99	84	**1,743**	**224**	5	37	5	219	**633**	8	188	770	2,451	6,466
	(2%)	(1%)	**(27%)**	**(4%)**	(0%)	(1%)	(0%)	(3%)	**(10%)**	(0%)	(3%)	(12%)	(38%)	(100%)

surg, surgery; radio, radiotherapy; chemo, chemotherapy; further details about ‘other therapy’ are not available; other treatment, other combinations of therapies; bold type, the three most frequent treatments of each cancer site; due to rounding error, row percentages do not always add up to 100 percent.

## Discussion

The objective of this study was to find out if the shared risk factor tobacco smoking leads to a significantly increased risk of TRSPC among persons with TRFPC compared to the general population. Furthermore, we tried to gain new insights into the possible etiology of the evaluated cancer types.

Comparisons between our own results and results from other research groups can be difficult because of different employed classifications of diagnosis groups. Hence, a one-to-one comparison is not always possible. All studies discussed in this section were registry-based and gave risk estimates for all subsequent cancers.

**Figure 1:**
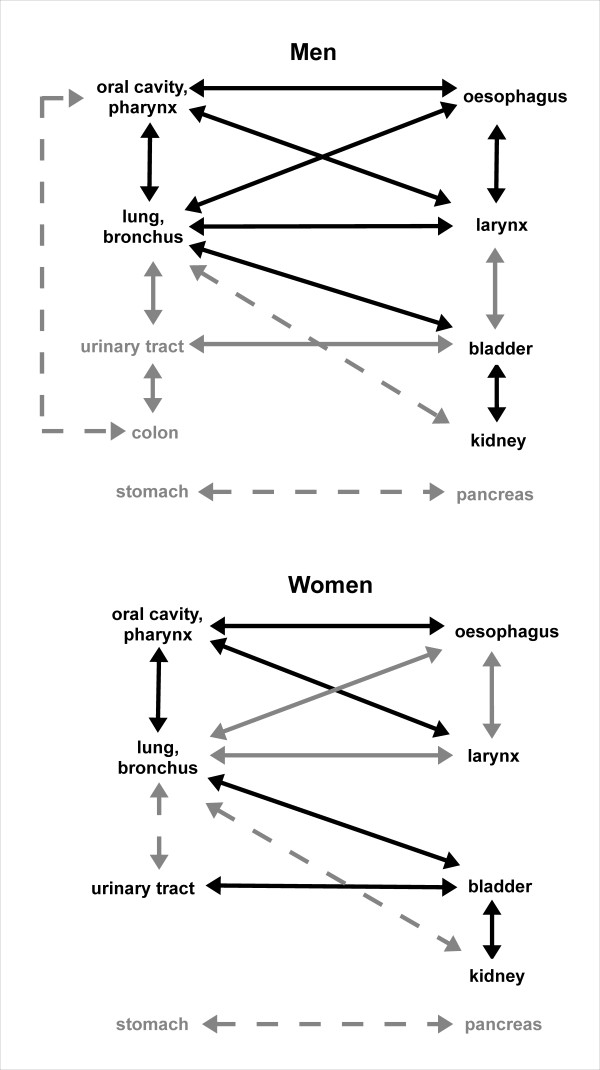
**Comparison between own results and SEER results.** Groups of tobacco-related malignancies with mutually and significantly increased risk of subsequent malignancy according to sex. Significant associations found both by SEER and in Bavaria are highlighted in black; associations with grey arrows are only found either in SEER results (solid arrows) or in own results (dashed arrows).

The SEER Program (Surveillance, Epidemiology and End Results Program) is the only available study that has analysed the risk of subsequent cancer in more than 50 cancer types in adults, that is based on a long observation period (1973–2000), and on a large population of more than 2 million cancer survivors [22]. With regard to tobacco-related malignancies, the results of SEER [17-21,23] are summarized in Figure 1 by identifying groups with mutually and significantly increased risk of TRSPC on the one hand, and by comparison with our own results on the other hand. 

In contrast to our study, colon and rectum cancer were separately evaluated, and lip cancer was excluded from the evaluation of first primary cancers in the oral cavity/pharynx, respectively, in the SEER study. However, in subsequent malignancies, the SEER study provided results on cancer of the oral cavity/pharynx including lip cancer (as was done in our study). Consequently, with respect to corresponding results, we can only perform a crude comparison in the aforementioned two cancer sites.

In contrast to our results, based on the SEER results, a group of malignancies can be identified in both genders consisting of the oral cavity/pharynx, oesophagus, lung/bronchus, and larynx. In addition to tobacco smoking, low fruit and vegetable intake is considered to be a shared risk factor for these cancer types (Table 3).

Tobacco smoking accounts for about 60% of all lung/bronchus cancer cases in women and for 90% of all lung/bronchus cancer cases in men [24]. The relative risk of lung/bronchus cancer among current smokers is 33 among men and 20 among women compared with lifelong non-smokers [25]. In our study, the diagnosis group of lung/bronchus showed a mutually significantly increased risk with almost every other diagnosis group. Therefore, with the exception of pancreatic cancer, stomach cancer, colorectal cancer, and leukemia, tobacco smoking can be confirmed as a shared risk factor in all tobacco-related cancer types considered in this study. The lack of mutually significantly increased relative risks between cancer in the oesophagus, larynx and lung/bronchus among women in our study might be due to insufficient sample size.

In both sexes, the group of urinary bladder, kidney and lung/bronchus was only found based on our own results, because in the SEER study, no mutually and significantly increased risk of SPC between the kidneys and the lung/bronchus was identified. However, tobacco smoking is the only known shared risk factor of these cancer types (Table 3). According to SEER, tobacco smoking accounts for about 50-65% of cases of urinary bladder cancer among men and for 20-30% of cases among women, respectively [17]. Furthermore, the hazard ratio of developing bladder cancer is 3.9 (95% CI, 3.5-4.4) among current male smokers and 4.7 (95% CI, 3.7-5.8) among current female smokers compared to never smokers [26].

The lack of a mutually significantly elevated risk of SPC between the urinary tract and bladder in men with regard to our own results may possibly be explained by the fact that in former decades, the misuse of phenacetin-containing analgesics (Table 3), a known risk factor for urinary cancer, was more common in European women than in men [27]. This fact might also explain the larger SIR with respect to these cancer sites in women compared to men (Table 2).

According to both the results of SEER and of the Bavarian Cancer Registry, the colon was a member of a group with increased risk of TRSPC in men but not in women. With regard to the results of SEER, the colon was a member of a dyad with the urinary tract, whereas in our study the colon/rectum formed a dyad with the oral cavity/pharynx. Based on the results shown in Figure 1, smoking does not appear to be a major risk factor in colorectal cancer. According to published reports, the multivariate-adjusted rate ratio of colorectal cancer mortality for current compared with never smokers is 1.3 (95% CI, 1.2-1.5) among men and 1.4 (95% CI, 1.3-1.6) among women [28]. Cigarette smoking accounts for only about 12% of colorectal cancer deaths [28]. Moreover, it is surprising that the dyad colon and oral cavity/pharynx was not found in the SEER study because of the additional shared risk factor low fruit and vegetable intake (Table 3) which may hint to differences between both countries with respect to the extent of this issue [29,30]. However, in our study, but not in the SEER study, the rectum was an additional member of this diagnosis group which somewhat limits the interpretation of this comparison.

Among both men and women, an isolated dyad was identified consisting of primary cancers in the stomach and pancreas which was found only in Bavaria. A mutually significantly increased risk of subsequent maligancy between these two cancer types was found in our study. However, neither in our own study nor in the SEER study was any of these two cancer types included in any other significant association. Furthermore, tobacco smoking is not considered to be a strong risk factor for stomach cancer. Therefore, tobacco smoking was not confirmed as a main risk factor in stomach and pancreas cancer. This finding, however, does not rule out a possible weaker etiological association of these two cancer types with tobacco smoking. The second known shared risk factor obesity (Table 3) may possibly help to explain this significant association. This dyad, however, was not observed in the SEER study although obesity appears to be a larger problem in the United States than in Europe [29,30]. Furthermore, it should be noted that pancreatic cancer has a very unfavourable prognosis which in turn leads to small absolute numbers of subsequent cancers. Among persons with pancreatic cancer, the relative 5-year survival rate in Germany is only 5-7% among men and 3-8% among women [24]. Consequently, the precision of the respective risk estimates was very limited.

Based on Figure 1, the diagnosis group of leukemia is the only cancer type which was not included in any group of mutually increased risk of TRSPC. In line with this finding, only a weak etiologic association with tobacco smoking has been reported for this malignancy [23].

In contrast to our results, SEER identified several significantly *reduced* risks of TRSPC. According to SEER, a significantly decreased risk of SPC in the lung/bronchus and in the urinary bladder after pancreatic cancer was identified in men. The explanation used by SEER is that pancreatic cancer has a very poor prognosis which in turn prevents the development of larger numbers of TRSPC. However, we neither found significantly decreased nor increased risks with regard to the aforementioned tumour sequences in our results, although tobacco smoking is a confirmed risk factor for all three cancer types.

The most surprising SEER result, however, was a significantly reduced risk of SPC in the oral cavity/pharynx and in the lung/bronchus, respectively, after kidney cancer in men. This is surprising because tobacco smoking is assumed to be a shared risk factor of these three cancer types. By contrast, our results showed a slightly increased risk of SPC in the oral cavity/pharynx in men, and a significantly increased risk (men: SIR, 1.51; women: SIR, 2.06) of subsequent lung cancer, respectively, after kidney cancer.

Furthermore, in contrast to our results, significantly decreased risks of SPC in the stomach (SIR, 0.8; 95% CI[SIR], 0.7-1.0) and colorectum (SIR, 0.7; 95% CI[SIR], 0.6-0.9) were identified in men with lung cancer in Finland during the period 1953–1995 [31]. In women, the respective risks were also (but not significantly) decreased (stomach cancer: SIR, 0.7; 95% CI[SIR] 0.3-1.4); colorectal cancer: SIR, 0.9; 95% CI[SIR], 0.5-1.6). In Finland, lung cancer has typically been a cancer type of the lower social classes in men whereas it was more frequent in the higher social classes in women at least until the mid-1980s [31]. Moreover, in Finland, the highest incidence of stomach cancer has been found in the poorer social strata, whereas colorectal cancer has typically been a cancer of the affluent classes [31]. These findings might possibly explain the observed significantly reduced risk of colorectal cancer among male patients, the decreased risk of stomach cancer among female patients, and the slightly decreased risk of colorectal cancer among female patients. An additional explanation of decreased risks might be the unfavourable prognosis of lung cancer (similar to pancreatic cancer) which in turn prevents the development of larger numbers of TRSPC in diseased persons.

Radiotherapy was administered in at least 42% of persons with FPC in the mouth/pharynx (Table 4). Furthermore, a considerable proportion of patients with cancer in the oesophagus (at least 24%), larynx (at least 29%), and lung/bronchus (at least 25%) was also treated with radiotherapy. Therefore, radiotherapy might have contributed to the significantly increased risk of TRSPC in these patients.

Since chemotherapy was applied in a relatively high proportion of persons with FPC in the mouth/pharynx (at least 23%), oesophagus (at least 29%), stomach (at least 18%), colon/rectum (at least 24%), pancreas (at least 39%), lung/bronchus (at least 39%), and leukemia (at least 41%), it might also have contributed to the significantly increased risk of TRSPC.

In comparison with our results, surgery was generally administered in a higher proportion of persons according to published German studies [32] (Table 5). These discrepancies may be due to the different observation periods. During the last decade, there seems to have been a tendency to conduct more clinical studies compared to former decades. This may have lead to a decreased proportion of exclusive surgical treatment and to an increased proportion of other treatments, which in turn may help to explain the observed differences with respect to treatment of cancer in the stomach, colon/rectum, pancreas, and urinary bladder. 

**Table 5:** **Cancer treatment according to site of first primary cancer in Germany (Munich Cancer Registry [1977–1993] unless mentioned otherwise; data sources,**[32,33])

	**Treatment of first primary cancer**									
**Site of first primary cancer**	**Only surg**	**Only radio**	**Only chemo**	**Surg + radio**	**Surg + chemo**	**Surg + other therapy**	**Radio + chemo**	**Surg + radio + chemo**	**Other therapy**	**Row sum**
mouth	**32%**	**13%**	3%	**32%**	1%	/	**13%**	6%	/	100%
oropharynx	10%	**21%**	2%	**37%**	1%	/	**19%**	12%	/	100%
oesophagus	**33%**	**21%**	/	12%	/	/	**20%**	/	14%	100%
stomach	**90%**	**/**	**/**	/	**/**	/	/	/	**10%**	100%
colon	**92%**	/	/	/	**8%**	/	/	/	**/**	100%
rectum	**80%**	/	/	**9%**	5%	/	/	**7%**	**/**	100%
pancreas	**100%**	**/**	**/**	/	**/**	/	/	/	/	100%
larynx	**38%**	**20%**	/	**37%**	/	/	/	5%	**/**	100%
non-small cell trachea/bronchus/lung	**40%**	**25%**	6%	/	/	/	**/**	/	**30%**	100%
small cell trachea/bronchus/lung	6%	**7%**	**46%**	/	/	/	**/**	/	**41%**	100%
kidney	**100%**^**12**^	/	/	**/**	/	**/**	/	/	/	100%
urinary bladder	**100%**^**3**^	/	/	/	/	/	/	/	/	100%
leukemia	/	/	**100%**^**14**^	/	/	/	/	**/**	/	100 %

surg, surgery; radio, radiotherapy; chemo, chemotherapy; bold type, the three most frequent treatments in each cancer site; ^1^treatment recommended by Deutsche Krebsgesellschaft (2008); ^2^treatment with curative intent only; ^3^invasive cancer only; ^4^including hematopoietic stem cell transplantation; due to rounding error, row percentages do not always add up to 100 percent.

Moreover, kidney cancer was most frequently treated with surgery alone, and leukemia was predominantly treated with chemotherapy (Table 4). Consequently, adherence to the recommendations of the Deutsche Krebsgesellschaft can be concluded with respect to the aforementioned two cancer types [33].

In summary, despite some differences, the main results of our study are confirmed by published international studies. With the exception of pancreatic cancer, stomach cancer, colorectal cancer, and leukemia, we were able to confirm the hypothesis of a significantly increased risk of TRSPC among persons with TRFPC compared to the general population. Hence, the aforementioned finding was most prominent in cancer types which are known to be strongly related to tobacco smoking.

The smoking prevalence of adults aged between 25 and 69 years in Germany has decreased in men and increased in women since 1986, but in the year 2006, the proportion of smokers among men (36%) was still higher than among women (28%) [34]. Due to the comparison with the general population in which the proportion of female smokers, and thus the risk of developing a tobacco-related cancer, is smaller compared to the study population, the relative risk of TRSPC among women with TRFPC compared to the female general population may be higher than that among men. Therefore, the aforementioned sex difference with respect to smoking prevalence might be the cause of higher SIR`s among women than among men in several tobacco-related cancer sites. However, there are known sex differences with regard to the attributable fractions of tobacco smoking in tobacco-related cancers. Therefore, this explanation is difficult to verify without knowledge of the individual smoking status in our study population.

The importance of smoking cessation programs for cancer patients with a TRFPC is underlined by results of a study of the National Cancer Institute (NCI) in Maryland revealing that 12 out of 55 (22%) lung cancer patients continued to smoke for at least six months after the diagnosis of lung cancer [35]. In view of the long-term reduction of tobacco-related cancer incidence, a rigid tobacco control policy should be advanced in Germany to further reduce the smoking rate [36]. The efficacy of a rigid tobacco control policy has previously been confirmed in all age groups [37]. Furthermore, lung cancer patients who stop smoking after the diagnosis of cancer show a steadily decreasing risk of TRSPC over time [35]. Since the relative 5-year survival rate of patients with cancer of the oesophagus, pancreas, and lung is below 23% in Germany [24], the efficacy of a rigid tobbacco control policy especially with regard to these cancer types would be considerable. By contrast, primary cancers in the kidney, urinary tract, and urinary bladder have a better prognosis with 5-year survival rates above 64%.

### Limitations of our study

Despite the large underlying data base obtained from a population-based cancer registry with an extensive catchment area, a number of limitations apply.

Firstly, no data on tobacco smoking among cancer patients was available. Consequently, an unknown degree of confounding can be assumed with regard to the obtained risk estimates which should be expected to differ considerably between smokers and non-smokers.

Secondly, only crude data about the applied therapies was available. For this reason and because the numbers of cases in each treatment stratum were too small to permit the calculation of meaningful results, we did not evaluate the risk of SPC stratified by treatment in order to evaluate possible risk differences. Likewise, calculation of the risk of SPC according to different histological tumour types was not possible due to insufficient numbers of events. Therefore, future studies should evaluate the risk of TRSPC according to different treatments and histological tumour types, respectively.

Thirdly, registration completeness of tobacco-related cancer cases was intermittingly below 90% in some parts of Bavaria during the observation period. In the year 2002, for example, the registration completeness among different diagnosis groups ranged between 67 and 99%. In general, a registration completeness of at least 90% is required to obtain valid results from population-based cancer registries [38]. This deficiency, however, was compensated in part by the employed standardisation of risk estimates based on the Bavarian general population, thus limiting the extent of severe risk underestimation. Therefore, the degree of confounding caused by this deficiency appears to be acceptable.

Fourthly, the overall duration of the present study was only seven years. However, Bavaria is the federal state (Bundesland) with the second largest population in Germany. Therefore, we consider the overall study population of 197,517 cancer patients and 331,759 person-years at risk to be sufficient for valid analyses. Furthermore, tobacco-related carcinogens can be assumed to have acted for several decades in many smokers prior to the diagnosis of TRFPC [39]. Therefore, in many smokers, TRSPC can be expected to develop within a relatively short time span (less than 10 years) after TRFPC. Consequently, we were able to evaluate the short-term risk of tobacco-related SPC according to 11 sites of first primary cancer.

## Conclusions

In conclusion, with the exception of pancreatic cancer, stomach cancer, colorectal cancer, and leukemia, the hypothesis of a significantly increased risk of TRSPC among persons with TRFPC compared to the general population was confirmed in all cancer types considered in this study. Furthermore, with regard to these cancer types, smoking was confirmed as a shared risk factor.

Consequently, our results confirm the dramatic consequences of tobacco smoking with regard to tobacco-related SPC. The results underline the importance of smoking cessation (preferably within special programmes) particularly among smokers with tobacco-related FPC in order to reduce the risk of multiple cancers. Furthermore, the results of this study illustrate the importance of follow-up examinations in smokers with tobacco-related FPC who continue to smoke in order to detect subsequent malignancies at an early stage. However, the calculation of the optimum duration and intensity of follow-up care among these patients requires further research.

## Competing interests

The authors declare that they have no competing interests.

## Authors' contributions

UB wrote the software, carried out the analysis, and drafted the manuscript. MRT participated in the development of the concept of the analysis and in the interpretation of results, revised the manuscript, and revised the English translation of the manuscript. MM revised the manuscript critically for important intellectual content. All authors have read and approved the final manuscript.

## Pre-publication history

The pre-publication history for this paper can be accessed here:

http://www.biomedcentral.com/1471-2407/12/250/prepub
